# SUPPORTING RESIDENTS WITH DEMENTIA LIVING AND DYING IN LONG-TERM CARE AND THEIR FAMILIES DURING COVID-19

**DOI:** 10.1093/geroni/igac059.940

**Published:** 2022-12-20

**Authors:** Sharon Kaasalainen, Abigail Wickson-Griffiths, Rose McCloskey

**Affiliations:** McMaster University, Hamilton, Ontario, Canada; University Of Regina, Regina, Saskatchewan, Canada; University of New Brunswick, Fredericton, New Brunswick, Canada

## Abstract

TThis presentation will share research findings about experiences during COVID-19 about implementing a virtual palliative toolkit in long-term care in Canada. The toolkit includes tools and practices to: (a) engage residents and families with dementia within a palliative approach to care, (b) develop workforce capacity through online education modules, (c) reduce stress and improve psychological health of residents, families, and staff, and (d) develop organizational structures and processes to promote a palliative approach to care. Individual interviews were conducted with residents, family members, and staff before implementing a palliative toolkit and after using it. Findings highlighted the negative impacts of COVID-19 on resident health due to isolation within home, preventing family from being at the bedside and cancelling stimulatory activities especially at end of life that were exacerbated by the lack of resources and government supports. Families appreciated the virtual supports and stated that they helped prepare them for their loved ones’ death while feeling more empowered, engaged, and supported in their journey. Although feedback from families was mostly positive, stating the virtual toolkit improved accessibility to information and supports, it was clear that some misunderstood terms, particularly what a palliative approach to care means; and others had challenges navigating the virtual platform to use the toolkit. Future work is needed to make the virtual tools more user-friendly so that they can be scaled up more widely.

